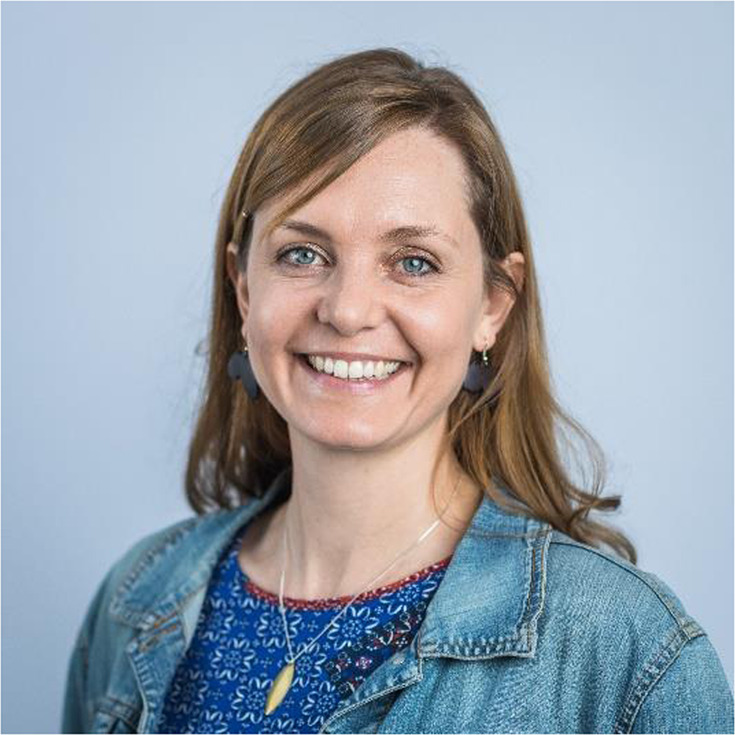# Finding my why

**DOI:** 10.1038/s41434-021-00242-0

**Published:** 2021-03-12

**Authors:** Samantha Barichievy

**Affiliations:** Discovery Sciences, BioPharma, AstraZeneca, Gothenburg, Sweden

**Keywords:** Diseases, Endocrine system and metabolic diseases

## The turning point

I fell in love in Toronto. Well, *with* Toronto. It was the first time I had travelled overseas on my own, or flown so far from home. The bus journey from the airport took me along tree-lined streets where the spring green of the new leaves was so bright and so crisp that I was sure my jet lagged brain must have mistakenly processed their natural hue for something surreal. The Ontario lake was in my peripheral vision the entire time, and it was so vast my mind kept defaulting to the thought of it as an ocean. The bus driver shouted out that my stop was next so I got off, looked around and was struck by the smell of humid wet air tinged with the intoxicating scent of freshly cut grass. And then I could not breathe. And my heart started beating so fast in my chest that I was sure it was going to give a final manic squeeze and that I would die right there on a pavement halfway across the world from my loved ones and my life.

I was in Toronto to give a talk at one of the biggest HIV conferences held every 2 years, and the city was awash with delegates, sponsors, and activists. I was a Ph.D. graduate at the time and wrapped up deeply in the world of how HIV manipulates the ancient and innate RNA interference pathway in human cells in order to create an optimal environment for itself. My advisor secured funding that allowed me to fly from the southern tip of Africa across the planet to mingle with a slice of the HIV community and broaden my network in an effort to open up my options for doing a postdoc somewhere in the developed world. Hard work had gotten me here. But so too had the immense fortune of simply being born into a middle class white family in South Africa. And an incredible amount of good luck. I had received several bursaries over the years that had eased the fiscal pressure of putting me through tertiary education in a country where a significant number of people face daily difficulties in affording basic resources. Coupled with my deep curiosity for figuring out how pathogens make us ill, the HIV burden in our country presented a clear path for my career as a scientist, although I was still unsure what my next step would be.

## Early career steps

Wits University was my intellectual home for 10 years straight out of school, starting with a BSc undergraduate degree. Growing up, I had consistently heard that ‘the world was my oyster’ largely because my predilection for reading meant I was good at book learning and, and subsequently excelled at formal education. The adage was unhelpful though as it gave me no steer, and instead when entering university I chose topics to study that were interesting in themselves, or taught by interesting people. Using such a gut-feel approach landed me in a new Molecular Medicine course for Honours and MSc degrees, which was crucial to getting me into a Ph.D. program based at Wits Medical School. Here the interactions with physicians, medical students and scientists helped me to silence a nagging voice in my head insisting that medicine was my true calling. A month shadowing a doctor friend in an emergency room at one of the biggest hospitals in the southern hemisphere reinforced a self-belief that I have a great capacity for resilience, but also that my compassion was better actioned through uncovering the mechanisms of infection and finding new medicines as opposed to being a medical doctor. Once again, my gut led me in the best direction although it wasn’t until long after I stopped breathing on the shores of lake Ontario that I understood why.

At no point in my 10 years journey did anyone sit me down and suggest a career path outside of academia. When I started my BSc I made up my mind to carry on until I had a Ph.D. because that’s the path my professors had trodden before me. My family and non-science friends were always supportive and encouraging of my choices, but their subject-naivety wrapped up with their vicarious enthusiasm for my studies meant I was not pushed to consider other options. I had a student’s perspective of the challenges faced by academics in a developing country, where one could tip the funding game in their favour by sticking to the big disease areas, use the ‘3rd world nation’ label to obtain reduced conference attendance fees, and stretch budgets in creative ways to provide what was needed to do great science. A critical edge in my training came from this environment, borne from a relative lack of funding compared to the big international labs—we simply did not have money to buy ready-to-use kits, and often had to re-use plasticware and make our own reagents to run experiments. Trouble-shooting and innovative solution finding were regular challenges, but combined with an almost obsessive leveraging of my scientific network, meant I still generated enough good data to end up with an oral presentation spot in Toronto, and an as yet unappreciated set of skills that were going to steer me right off my tidy and conventional academic career path.

## Piled higher and deeper

I think I suffered the same mental exhaustion and self-identity crisis as Ph.D. graduates the world over. Mine had a dramatic moment on the shores of Lake Ontario where I suffered a panic attack on my own, comforted by a stranger who stopped to ask if I was okay and offered me a hug. My talk went well and I increased my scientific network, but the lake-side incident started me thinking about what I could do to avoid experiencing anything similar again. I was sure the deep anxiety I felt was connected to the choices I was considering for my next career move, but I felt lost. Back home in South Africa I reached out to a cherished colleague who was a celebrated scientist, wife and mom for advice on what to do next. I applied for and won a Sydney Brenner Postdoctoral Fellowship and so began what I was sure would be the last key step in me setting up my own independent research group. My postdoc advisor assembled an incredible team of people and together with them I was challenged daily in every interaction and on every topic. The pace was relentless, the science fascinating, the learning curve steep, and the panic attacks regular. Towards the end I naturally shifted focus to where and how I could start my own group, underpinned by a burning drive to do so in a way that would dial down my anxiety levels.

## Getting off the tidy path

It took 5 years of failed applications, countless tweaks to my resume, numerous rejection letters, and reading some books that specifically and dramatically altered my thinking, but I found my place. Industry. Pharma to be precise. And in a location halfway across the planet. My husband and I were unbelievably lucky to end up in the same undergraduate course learning how to streak bacteria onto agar plates, and make our own Luria broth. He had coached and counselled me through all degrees and panic attacks, while we journeyed together through our studies, out into mainstream science with our Ph.Ds and wedding rings. Balancing two similar science careers was tricky, even more so in a relatively small community with no biotech or industry opportunities at all. We wanted each other as our happiest selves, a family, impactful careers doing something interesting, and an outdoor life to balance everything—in that order. Once we had decided that, our choices narrowed on Scandinavia and so began ‘the failure years’. Asking for candid feedback after each rejection was critical to helping hone my CV and interview skills, and led me to a tough yet ultimately successful application to join AstraZeneca in a new team tasked with improving the success rate of early drug discovery. Resilliance and perserverance were also integral in helping me to rebound from each failure, but focusing on what to change for the next round and thus apply the learnings in a new way, helped reframe things more positively. And so, 5 years on, we traded Johannesburg in South Africa for Johanneberg in Sweden, and began an entirely new life for ourselves.

## Steps towards finding purpose

Managing people and leading people are two, sometimes interwoven but really distinct, skill sets. I was again extremely lucky to spend time with research group leaders who managed their teams through listening, coaching, and providing opportunities for development in an arena that also fostered constructive criticism. However it was not until I joined AstraZeneca that I understood the power of excellent people management. We run parallel career tracks covering both a scientific and management ladder with the possibility to move between the two. Everyone is responsible for their own career development which is formalised through annual goal setting, and regular discussions where our gaps and strengths are identified, and then actively addressed. Opportunities abound and often it feels that the only downside is the seemingly endless choice. I joined the company as a scientist in a team supervised by a great manager. He set me on a path for rapid advancement and through specific, guided nurturing enabled me to leap two career levels in 4 years, while navigating the massive jump from academia to industry. He gave me candid advice, introduced me to mentors who still steer me, and challenged me to consider where I could make my greatest impact in the company and for myself. I used these interactions and introductions to seek out advice on an array of topics, all the while listening and learning with ears and eyes wide open in this wonderful environment.

6 years on, I am happy, fulfilled, balanced and calm. I manage a team of incredible scientists working to build different molecular tools for understanding disease. We use them to uncover key targets that can be manipulated to shift a disease balance back in our favour. It’s fantastic work—it’s extremely challenging technically and intellectually, it’s a highly collaborative effort, and our curiosity is rewarded by the potential of improving someone’s health. Part of this involves gene therapy which is emerging worldwide as a powerful tool for tackling diseases at the most fundamental level—their genomes. Jennifer Doudna and Emmanuelle Charpentier, along with an army of collegues, have provided us with a game-changing engine that we can exquisitley tweak in order to drive certain disease outcomes in favour of health. With women like them as guiding stars, ample luck and my own grit, I have found my truest career purpose yet, and feel remarkably grateful to be in such a nourishing environment. Our choice to move to Sweden means we have time for both our careers and home life without sacrificing either—it’s simply baked into the DNA of Swedish society. We also consciously chose to move here and raise a family, and are doing so as a unit of four without having felt that it impinged on our career trajectories. I spent 2 full years at home on parental leave during my 6 at AstraZeneca, and still advanced in a way that I am not sure is possible in many other locations. We sacrificed the geographical closeness of family and friends with history, yet feel included and settled both professionally and personally, making the hurt caused by distance manageable. It’s unlikely that I would tell a 20 year old version of myself not to follow the same path, as I am so pleased with where I am now. However I’d tell her not to fester in the negativity that settles after every panic attack, as they can bring joy down the line if you are open to listening, revisit failures to improve for the next time, and have a lot of luck.